# Supplementary material for: Baseline Characterization of the Gut Microbiota of Field and Colony Populations of Phlebotomus tobbi and Preliminary Assessment of the Anti-Leishmanial Activity of Cultivable Bacteria
Source: Pathogens. 2026 Jun 23;15(7):658. doi: 10.3390/pathogens15070658 (PMC13416292; doi:10.3390/pathogens15070658)
Supplement: Supplementary file 1 [file pathogens-15-00658-s001.zip › pathogens-4357168-File S1/Taxa2ASV_Decomposer/Family/f__Burkholderiaceae/heatmap_with_clustering.pdf]

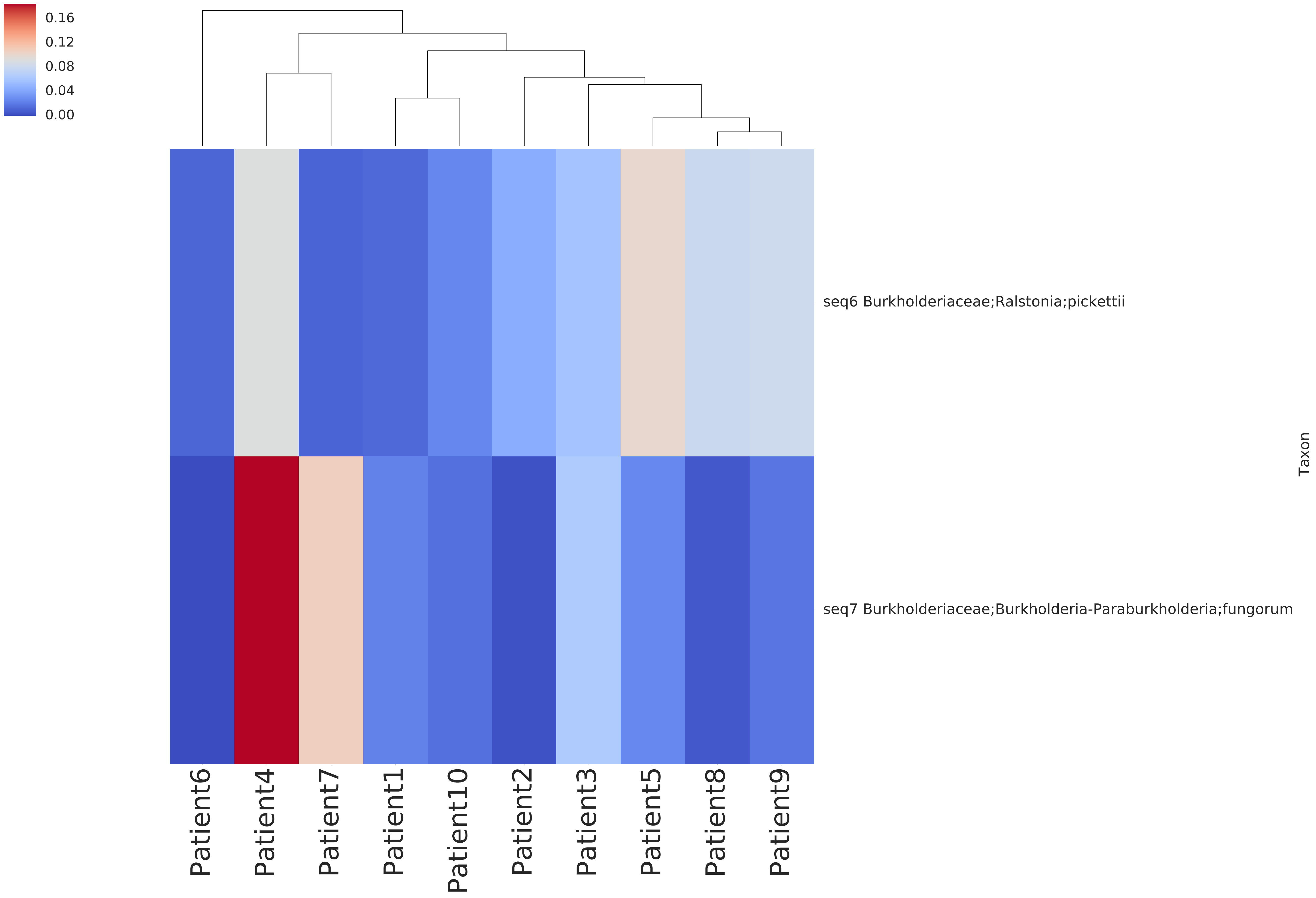
0.16
0.12
0.08
0.04
0.00
seq6 Burkholderiaceae;Ralstonia;pickettii
seq7 Burkholderiaceae;Burkholderia-Paraburkholderia;fungorum
Taxon
Patient6
Patient4
Patient7
Patient1
Patient10
Patient2
Patient3
Patient5
Patient8
Patient9
